# Clinical pharmacology of tramadol and tapentadol, and their therapeutic efficacy in different models of acute and chronic pain in dogs and cats

**DOI:** 10.5455/javar.2021.h529

**Published:** 2021-09-19

**Authors:** Adriana Domínguez-Oliva, Alejandro Casas-Alvarado, Agatha Elisa Miranda-Cortés, Ismael Hernández-Avalos

**Affiliations:** 1 Universidad Autónoma Metropolitana, Mexico City, Mexico; 2 Department of Biological Sciences, Clinical Pharmacology and Veterinary Anesthesia, Faculty of Higher Studies Cuautitlán, Universidad Nacional Autónoma de México, Mexico City, Mexico

**Keywords:** Analgesia, cats, dogs, pain, tapentadol, tramadol

## Abstract

Opioids are considered the gold standard to manage acute or chronic or mild to severe pain. Tramadol is a widely prescribed analgesic drug for dogs and cats; it has a synthetic partial agonism on μ-opioid receptors and inhibits the reuptake of norepinephrine and serotonin. However, the biotransformation and resultant metabolites differ between species and depend on cytochrome P450 interactions. Dogs mainly produce the inactive N-desmethyl tramadol metabolite, whereas cats exhibit an improved antinociceptive effect owing to rapid active O-desmethyltramadol metabolite production and a longer elimination half-life. Tapentadol, a novel opioid with dual action on μ-receptors and noradrenaline reuptake inhibitory activity, is a promising option in dogs, as it is less reliant on metabolic activation and is unaffected by cytochrome polymorphisms. Although scientific evidence on the analgesic activity of tapentadol in both species remains limited, experimental studies indicate potential benefits in animals. This review summarizes and compares the pharmacology, pharmacokinetics, and therapeutic efficacy of tramadol and tapentadol in dogs and cats with different pain conditions. According to the available data, tramadol seems a more suitable therapeutic option for cats and should preferably be used as a component of multimodal analgesia in both species, particularly dogs. Tapentadol might possess a superior analgesic profile in small animals, but additional studies are required to comprehensively evaluate the activity of this opioid to manage pain in dogs and cats.

## Introduction

Analgesia is an essential aspect of veterinary patient care that prevents pathophysiological complications owing to pain, reduces the stress response during surgical procedures [1], and improves overall animal welfare [2]. To select an appropriate analgesic therapy, the etiology, type, severity, and chronicity of the process must be considered to prevent central and peripheral sensitization in response to a noxious stimulus [3–5]. Currently, multimodal analgesia is considered the most appropriate strategy for pain management [6,7], and commonly used therapies include non-steroidal anti-inflammatory drugs (NSAIDs) [8], non-opioid NSAIDs such as paracetamol [9], local anesthetics [10], alpha-2 adrenergic agonists [11], N-methyl-D-aspartate (NMDA) antagonists [12], or opioids like tramadol and tapentadol [13].

In general, opioids are considered the gold standard to manage mild to intense, short, or long-term pain and represent a perioperative protocol to improve recovery times and decrease post-surgical pain scores [14]. They act on central and peripheral opioid receptors (primarily, G protein-coupled receptors), cause hyperpolarization of the neurons, and lessen the secretion of excitatory neurotransmitters to interrupt the transduction, transmission, modulation, and perception of a noxious stimulus [13].

Tramadol and tapentadol are central non-traditional opioids with dual effects [14]. Tramadol is a synthetic opioid agonist at μ-opioid receptors (MOR) that suppress serotonin (5-HT) and norepinephrine (NE) reuptake [15]. It acts primarily on the descending inhibitory pathway but requires frequent dosage intervals and concomitant administration with other drugs to maintain adequate therapeutic efficacy [14]. Conversely, tapentadol is a recently developed drug with the earliest evidence documented in 2010 [16]. This opioid is considered the first MOR – norepinephrine reuptake inhibitor (NRI) agent – given its combined agonist action on MORs and the potent inhibition of NE reuptake [17]. The analgesic efficacy and therapeutic safety of these drugs depend on the delivery method, gene diversity between species, and their association with other analgesics [18]. In addition, their use in small animals remains controversial due to discrepancies between clinical and experimental outcomes [13].

In the present review, we aim to summarize the pharmacokinetics, pharmacodynamics, main adverse effects, contraindications, and therapeutic efficacy of tramadol and tapentadol in dogs and cats during different acute and chronic pain models. Through Web of Science, PubMed, Google Scholar, Scopus, and Elsevier, relevant keywords regarding the administration of tramadol and tapentadol to manage pain in dogs and cats were used to identify and select publications associated with these opioids. The chosen publications covered the following inclusion criteria: 1) articles after 2000 where tramadol or tapentadol pharmacokinetics was first reported; 2) reports from 2010 to 2021 where tramadol and tapentadol were used in a clinical setting as analgesic therapy for different surgical procedures or painful conditions; and 3) studies where their antinociceptive properties were tested in dogs or cats, or current animal models.

## Clinical Pharmacology of Tramadol

Tramadol is an atypical opioid with a weak action on μ-receptors and modulates the descending noradrenergic and serotonergic pathways by inhibiting the reuptake of the monoamines (5-HT and NE), as well as 5-HT release [19]. In addition, this opioid is known to possess mechanisms such as an agonist activity at TRPV1 receptors, inhibition of G protein-coupled receptors (alpha-2 adrenoceptors, neurokinin receptor 1, and muscarinic receptors), inhibitory effects on nicotinic acetylcholine receptors, and NMDA receptor antagonism [13,20]. These characteristics mediate the opioid and non-opioid mechanisms of action [21]. Given its rapid onset of action (10–20 min), it is frequently employed in small animals to manage moderate to severe perioperative pain [22] and osteoarthritic diseases [20].

Surveys conducted in Colombia [23] and Brazil [24] have revealed that tramadol is the most commonly prescribed opioid in small animals (more than 80%) [23], corresponding to 58% and 50% of preoperative analgesic management in dogs and cats, respectively, and 62% and 53% in terms of post-surgical therapy. On the contrary, in Canada, the United States, and Europe, butorphanol and buprenorphine were reported as the most commonly used drugs. These distinct utilization patterns can be attributed to the availability of tramadol as an over-the-counter drug in Brazil and Colombia [24].

According to the available literature, tramadol offers a better therapeutic range to control pain when co-administered with other drugs rather than monotherapy, and its pharmacokinetic properties considerably differ between dogs and cats and even within species. These differences are compared and presented in Table 1 [25–32] when administered by different routes and distinct doses. Table 2 [25–32] shows a comparison between half-life, peak concentration, and time peak of the main active *O*-desmethyltramadol (M1) metabolite in dogs and cats. Although data on cats and dogs has been previously reported, the pharmacokinetics of tramadol in other species such as koala has been recently determined and compared with those in dogs and cats following a subcutaneous (SC) dose of tramadol (2 and 4 mg/kg); the results revealed a half-life of 2 ± 1.03 h, which was similar to that in cats (3–4 h) [33].

Tramadol undergoes biotransformation by demethylation, oxidation, and hepatic conjugation by the uridine diphosphate (UDP) UDP-glucuronyltransferase (UGT) enzymes in phase II metabolism, forming at least 30 different compounds [18]. However, only three of these metabolites have analgesic activity: M1, N,O-didesmethyltramadol (M5) (a result of the metabolism of M1), and tramadol [34]. M1 is the principal active metabolite and the most relevant component that mediates the analgesic effects due to its high affinity to MOR (up to 200 to 300-fold greater than the parent compound) [16,19]. N-desmethyltramadol (M2) is another metabolite considered inactive and formed by the liver microsomes, mainly in dogs [35].

Tramadol is a mixture of two trans-enantiomers: (+) (1R, 2R)-tramadol and (−) (1S, 2S)-tramadol, which confers antinociceptive effects [36]. (+)-Tramadol has a greater affinity to MOR and is responsible for inhibiting 5-HT reuptake and its extracellular increase. Contrarily, (−)-tramadol is a potent inhibitor of NE reuptake [37]. (+)-M1 is an active metabolite with agonism to μ-receptor, while (−)-M1 inhibits NE reuptake [35]. Additionally, a greater association to MOR is observed on M5, compared to (−)-M1 and tramadol [18]. Accordingly, the binding of the (+)-tramadol stereoisomer and (+)-M1 to μ-receptors are pivotal for producing the analgesic effect [37]. Some reports have suggested that MOR agonism is predominantly effective during acute pain management. On the contrary, the inhibition of 5-HT and NE recapturing is appropriate during chronic pain states [18]. Moreover, compared to the inhibition of 5-HT reuptake, NE has a greater role during pain modulation [16].

**Table 1.** Comparison of mean ± SD values for some tramadol pharmacokinetic variables in dogs and cats.

Species	Route and dose	Parameters (units)	Mean ± SD values	References
Dogs	IV (4 mg/kg)	*F*% Vd (l/kg) Cl (ml/min/kg) *T*_max_ (h) *C*_max_ (ng/ml)	ND 3.01 ± 0.45 54.63 ± 8.19 ND ND	[25]
	IV (4 mg/kg)	*F*% Vd (ml/kg) Cl (ml/h/kg) *T*_max_ (h) *C*_max_ (μg/ml)	ND 1,003 ± 472 923 ± 460 ND ND	[26]
	IV (4 mg/kg) 2 years	Vd (l/kg) Cl (ml/min/kg)	4.77 ± 1.07 29.9 ± 7.3	[27]
	IV (4 mg/kg) 8–10 years	Vd (l/kg) Cl (ml/min/kg)	4.73 ± 1.43 23.7 ± 5.4	[27]
	IV (2 mg/kg)	*F*% Vd (ml/kg) Cl (ml/h/kg) *T*_max_ (h) *C*_max_ (μg/ml)	ND 1,995.89 ± 1,165.24 1,748.99 ± 1,239.6 ND ND	[28]
	EPI (2 mg/kg)	*F*% Vd (ml/kg) Cl (ml/h/kg) *T*_max_ (h) *C*_max_ (μg/ml)	82 ND ND 1.15 ± 0.31 0.18 ± 0.12	[28]
	IM (4 mg/kg)	*F*% Vd (ml/kg) Cl (ml/h/kg) *T*_max_ (h) *C*_max_ (μg/ml)	92 ± 9 293 ± 151 1,131 ± 146 0.34 ± 0.05 2.52 ± 0.43	[29]
	Oral (11.2 ± 2.0 mg/kg)	*F*% Vd (l/kg) Cl (ml/min/kg) *T*_max_ (h) *C*_max_ (ng/ml)	65 ± 38 ND ND 1.04 ± 0.51 1,402.75 ± 695.52	[25]
	Oral (5 to 7 mg/kg)	*F*% Vd (l/kg) Cl (ml/min/kg) *T*_max_ (h) *C*_max_ (ng/ml)	ND 5.4 30 3.54 195	[30]
	Rectal (4 mg/kg)	*F*% Vd (ml/kg) Cl (ml/h/kg) *T*_max_ (h) *C*_max_ (ng/ml)	10.03 ± 4.51 392 ± 221 10,420 ± 3,463 0.56 ± 0.41 140 ± 60	[26]
Cats	IV (2 mg/kg)	*F*% Vd (l/kg) Cl (ml/min/kg) *T*_max_ (min) *C*_max_ (ng/ml)	ND 3.0 ± 0.1 20.8 ± 3.2 ND 1,323 ± 92	[31]
	IV (2 mg/kg)	*F*% Vd (ml/kg) Cl (ml/h/kg) *T*_max_ (h) *C*_max_ (μg/ml)	ND 1,953.65 ± 418.68 895 ± 366.62 ND ND	[32]
	Oral (5.2 mg/kg)	*F*% Vd (l/kg) Cl (ml/min/kg) *T*_max_ (min) *C*_max_ (ng/ml)	93 ± 7 5.1 ± 0.3 18.6 ± 3.2 25 914 ± 232	[31]

*F*% = bioavailability, ND = not determined, Vd = apparent volumen of distribution at steady-state, Cl = clearance, *T*_max_ = time of maximum plasma concentration, *C*_max_ = maximum plasma concentration.

**Table 2.** Comparison of mean ± SD values for some *M1 pharmacokinetic variables in dogs and cats*.

Species	Route and dose	Parameters (units)	Mean ± SD values	References
Dogs	IV (4.4 mg/kg)	*λ*_*z*_ (1/h) *T*_max_ (h) *C*_max_ (ng/ml)	0.44 ± 0.12 0.43 ± 0.20 146 ± 40.51	[25]
	IV (4 mg/kg)	*λ*_*z*_ (1/h) *T*_max_ (h) *C*_max_ (μg/ml)	0.45 ± 0.39 0.94 ± 0.52 0.02 ± 0.01	[29]
	EPI (2 mg/kg)	*λ*_*z*_ (1/h) *T*_max_ (h) *C*_max_ (μg/ml)	3.77 ± 1.74 1.14 ± 0.72 0.20 ± 0.08	[28]
	IM (4 mg/kg)	*λ*_*z*_ (1/h) *T*_max_ (h) *C*_max_ (μg/ml)	0.34 ± 0.06 0.88 ± 0.18 0.6 ± 0.01	[29]
	Oral (11.2 ± 2.0 mg/kg)	*λ*_*z*_ (1/h) *T*_max_ (h) *C*_max_ (ng/ml)	0.33 ± 0.07 0.50 ± 020 449.13 ± 210.10	[25]
	Oral (5 to 7 mg/kg)	*λ*_*z*_ (1/h) *T*_max_ (h) *C*_max_ (ng/ml)	4.67 2.82 4.60	[30]
	Rectal (4 mg/kg)	*λ*_*z*_ (1/h) *T*_max_ (h) *C*_max_ (ng/ml)	0.45 ± 0.39 0.94 ± 0.52 20 ± 12	[26]
Cats	IV (2 mg/kg)	*λ*_*z*_ (min) *T*_max_ (min) *C*_max_ (ng/ml)	261 ± 28 55 ± 17 366 ± 31	[31]
	IV (2 mg/kg)	*λ*_*z*_ (h) *T*_max_ (h) *C*_max_ (μg/ml)	3.54 ± 1.17 0.25 ± 0.0 0.81 ± 0.23	[32]
	Oral (5.2 mg/kg)	*λ*_*z*_ (min) *T*_max_ (min) *C*_max_ (ng/ml)	289 ± 19 53 ± 13 655 ± 77	[31]

*λ*_*z*_ = plasma half-life, *T*_max_ = time of maximum plasma concentration, *C*_max_ = maximum plasma concentration.

**Figure 1.**
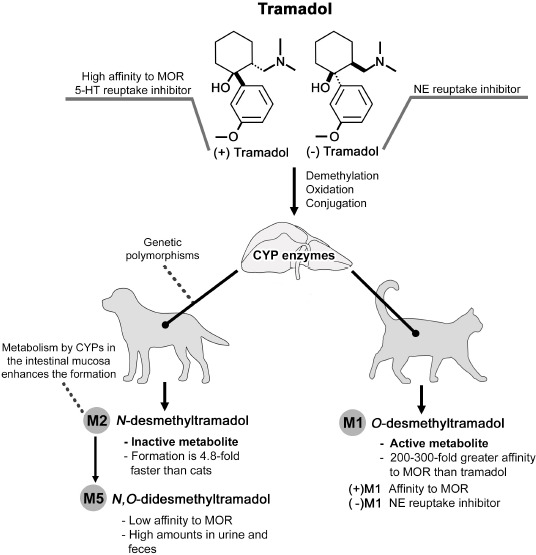
Pharmacokinetic differences of tramadol in dogs and cats [29,38].

In dogs and cats, the considerable variations in metabolite formation can be attributed to the cytochrome P450 (CYP) (CYP2D15 in dogs) inhibition and genetic polymorphisms [38] (Fig. 1). The CYPs constitute the main hepatic enzymes for drug biotransformation in human and non-human animals, and genetic variations reportedly influence clinical outcomes, given their impact on therapeutic effects of metabolism-dependent analgesics tramadol. The complete loss of a single protein that encodes a CYP or a single nucleotide polymorphism can alter the enzyme conformation, potentially reducing its enzymatic capacity. CYP2D is solely responsible for M1 production in dogs, and mutant alleles have been linked to differences between species and breeds [38]. For instance, in Bullmastiffs, Border collies, Rottweilers, and English cocker spaniels, van Hagen et al. [39] reported that all breeds demonstrate differences in coding sequences or exons 4, 5, and 6, but differences in exon 2 were exclusively detected in the Border collies. In other CYPs, such as the *CYP2C41* gene, the site of gene deletion was consistently absent in Bearded Collies, Boxers, Bernese Mountains, Briards, French bulldogs, and Irish Wolfhounds. On the contrary, the enzyme was present in breeds such as Chinese shar-pei, Siberian husky, Schapendoes, and Kangals. Mutations in CYP oxidoreductase (POR) are reportedly prevalent in breeds like Greyhounds, which can metabolize CYP2B11 substrates but not CYP2D15; this could be translated into an altered metabolism in all breeds related to Greyhounds [40]. Although *in vivo* and clinical trials to determine the pharmacokinetics of tramadol within breeds are limited, these genetic modifications are associated with poor antinociceptive effects in Beagles, as mentioned by Kögel et al. [21] and Schütter et al. [41]. However, not all polymorphisms result in enzymatic deficits, indicating that the capacity of the mutant CYP is not always reduced [39]. In the case of dogs, this implies that, based on the available data and inconsistencies between breeds, therapeutic dosing can be less efficacious in some dogs due to high enzymatic biotransformation, increased clearance, and minimal plasma concentrations [42], or could be attributed to a metabolically less active enzyme that produces fewer active metabolites [39].

Conversely, cats are known to alter the metabolism of medications, given their enzymatic deficiency in functional UGT (UGT1A6 and UGT1A9), N-acetyltransferase and thiopurine S-methyltransferase. This loss reduces or completely suppresses the CYP catalytic activity, resulting in slow clearance, a higher plasma concentration of some compounds, and an enhanced risk of developing more adverse effects and toxicity [42]. To date, it remains unknown which feline CYP enzyme is responsible for the conversion of tramadol to M1 [43]; however, an *in vitro* report by Izes et al. [44] revealed that the rate of depletion of M1 in dogs exhibited an intrinsic clearance (Cl_int_) of 22.8 μl/min/mg, whereas in feline microsomes, the concentration of M1 was not depleted owing to the lack of CYP2B-like metabolism in this species. Accordingly, cats demonstrate reduced glucuronidation activity in phase II due to the lack of CYP2B6, the enzyme participating in the metabolism of M1 and is present in humans and dogs, with CYP2B11 as an analog in canines. Therefore, the lack of CYP2B6 in cats and the complete absence of phase II glucuronidation in feline microsomes, along with the minimal depletion of M1 in phase I, may clarify why cats present higher M1 concentrations in the bloodstream. On the contrary, dogs not only produce less M1 but also demonstrate rapid metabolite conjugation owing to the activity of the CYP2B11 enzyme, a pathway unavailable to cats. Recently, Ono et al. [45] reported that the CYP2C subfamily revealed some pseudogenes in cats, and its hepatic and intestinal presence was negligible, limiting the biotransformation; in addition, fewer than one-third CYPs were found to be available for phase I metabolization in cats when compared with dogs [46]. However, although these routes are deficient in cats, other pathways such as sulfation, acetylation, and methylation can also contribute to the metabolism and elimination of tramadol [44].

Notably, the metabolism of tramadol relies on hepatic enzymes, and this influences the amount of M1 produced [35], particularly in dogs, where the generation of M1 is 3.9-fold slower than in cats, but the production of the inactive M2 is faster (4.8-fold) [38]. To enhance the bioavailability of tramadol in dogs, particularly in Beagles, a soft capsule containing acetaminophen and tramadol was developed and demonstrated better bioavailability in dogs; however, additional studies and its application in a clinical setting as an analgesic adjuvant are crucial to evaluate the therapeutic potential of this new formulation [47]. The metabolism via CYPP450s in the intestinal mucosa can also influence the high amounts of the inactive compound. Likewise, the oxidation of M1–M5, which has a lower potency for MOR and low central nervous system (CNS) penetration ability, is faster in dogs than in cats [38]. The main route for drug excretion (M1, M2, and M5) is via urine (90%), and a small amount undergoes biliary and fecal elimination (10%) [16].

Tramadol is commonly administered via oral and intravenous (IV) routes. IV administration is typically administered at 2–4 mg/kg, as a sole drug or as multimodal analgesia, while oral therapy ranges between 4 and 10 mg/kg/6 h in dogs and 1–4 mg/kg in cats [48]. In both dogs and cats, the route of administration reportedly influences certain pharmacokinetic parameters and the bioavailability of tramadol. For example, Di Salvo et al. [49] determined that intranasal administration in dogs (4 mg/kg) presented a half-life of 1.03 ± 0.53 h, a *T*_max_ 0.67 ± 0.22 h, and a *C*_max_ 123.17 ± 46.33 ng/ml, with an *F*% between 3.26% to 20.6%. Interestingly, despite the observed bioavailability, bitches undergoing elective surgery exhibited similar analgesia. On the contrary, extradural (ED) administration reached a bioavailability of 82%, a longer half-life (2.66 ± 0.50 h) and higher drug concentration of 0.18 ± 0.12 μg/ml [28]. Tables 1 and 2 summarize the values observed following administration via these different routes.

Regarding differences between species, tramadol presented a shorter half-life in dogs (1–2 h) following oral administration with 10 mg/kg [50]. KuKanich [51] suggests that canines require higher doses (15 mg/kg/6–8 h, orally); however, therapeutic concentrations remain difficult to attain. Furthermore, repeated dosing decreased absorption and plasma concentration [50]. Typically, high doses of tramadol are well tolerated in dogs; however, nausea, salivation, anorexia, and sedation are common side effects. These adverse reactions may result from 5-HT and NE reuptake rather than opioid-linked mechanisms [41]. Some reports have indicated that administration of 40 mg/kg/day for 1 year resulted in adverse effects such as mydriasis, reduced body weight, restlessness, difficulty walking, salivation, vomiting, tremors, and seizures [50].

Pharmacokinetic data suggest that, given the higher bioavailability and slower clearance, the half-life of tramadol and M1 is longer in cats (3–4 h for tramadol; 4 to 6 h for the metabolite) [51,52]. In addition, M1 concentrations following oral administration of 5.2 mg/kg are higher than those reported in humans; hence, oral doses of 1–2 mg/kg/12 h or 5–10 mg per cat every 12 h are considered appropriate [51]. Following IV injection, higher M1 formation has been detected due to its slow conjugation, and there are no reports of other metabolites found in cats [38].

Although tramadol has a broad safety in animals, cats can exhibit excitement, dysphoria, euphoria, and mydriasis, and accordingly, a lower dosage is recommended to avoid these manifestations [53]. In addition, the co-administration of drugs like famotidine or omeprazole may decrease the risk of gastrointestinal issues when tramadol is used with NSAIDs [50].

The effects of tramadol can be partially antagonized by naloxone, alpha-2 antagonists (yohimbine), and serotonin antagonists (ketanserin and ondansetron). Furthermore, acetylcholine could prevent the inhibitory action of M1 on muscarinic receptors [51]. The opioid, along with selective reuptake inhibitors, seems to increase its effectiveness; however, it should be noted that coadministration with selective 5-HT reuptake inhibitors such as fluoxetine and trazodone), monoamine oxidase inhibitors (e.g., selegiline), and tricyclic antidepressants (e.g., amitriptyline, clomipramine) can increase the risk of developing 5-HT toxicity, known as “serotonin syndrome” [18,48].

As highlighted, the variability of M1 concentrations between species indicates that tramadol is more suitable for cats than dogs. In addition, the extended elimination half-life and greater amounts of plasma M1 can be attributed to lower glucuronidation activity observed in cats [22,37], in contrast to dogs, who have reported plasma M1 levels below therapeutic ranges even when compared with humans [4].

## Therapeutic Efficacy of Tramadol in Dogs

In dogs, the subtherapeutic concentration of M1, given the abundance and type of liver enzymes necessary for its metabolization [48], implies that tramadol monotherapy is unlikely to have meaningful benefits for pain control in this species [51]. A recent meta-analysis published by Donati et al. [54] documented a substantially low certainty of the evidence in terms of its analgesic action for managing postoperative pain. However, the results of multiple studies using the surgical model of ovariohysterectomy (OVH) in bitches, a procedure where tramadol is commonly administered during the perioperative period or as rescue analgesia [55], revealed divergent conclusions.

For example, in one of the earliest reports where authors compared tramadol IV (2 mg/kg) to another well-known opioid (i.e., morphine at 0.2 mg/kg), they did not detect any differences in analgesia levels in bitches undergoing OVH [56]. In addition, the concentrations of catecholamines, cortisol, and glucose remained unaltered, and the postoperative pain scores were low in both groups. Although two animals treated with tramadol and only one treated with morphine required rescue analgesia, Mastrocinque and Fantoni [56] concluded that tramadol has a similar analgesic effect as morphine to control post-surgical pain. On the contrary, according to the Glasgow Composite Measure Pain Scale, nefopam IV at 2 mg/kg conferred superior analgesia than the same dose of tramadol, whereas patients receiving tramadol required less rescue analgesia (1 mg/kg IV of nefopam) [57]. Furthermore, Meunier et al. [58] reported that the preoperative IV application of 0.2 mg/kg meloxicam and 4 mg/kg tramadol as a multimodal analgesic regimen for free-roaming dogs subjected to OVH provided adequate analgesia but no clinical benefit over the use of meloxicam alone. Interestingly, the need for rescue analgesia was approximately four times greater in animals administered meloxicam than in the opioid group.

The SC route has been used in bitches both pre- and post-surgery (4 mg/kg) every 12 h to evaluate hematological and biochemical changes in pain stress after OVH [59], as well as following surgical treatment of pyometra (3 mg/kg every 6 h), as a multimodal approach along with SC meloxicam (0.2 mg/kg) [60]. Rossetti et al. [61] compared the analgesic effect of tramadol SC (3 mg/kg) and maropitant (1 mg/kg) during the post-surgical period in bitches. The authors reported that 5 out of 10 animals in the tramadol group and 6 out of 10 animals in the maropitant group required rescue analgesia with morphine, indicating that none of the drugs are appropriate as monotherapy to control immediate surgical pain. Conversely, epidural (EPI) opioids offer excellent long-term analgesia at a lower dose than systemic administration, as the opioid directly binds to receptors in the spinal cord, inhibiting the nociceptive signal and modulating the release of epinephrine and NE in descending pathways [62]. Mastrocinque et al. [62] compared EPI tramadol with intramuscular (IM) tramadol administration in elective surgery. The authors revealed that EPI was safe and afforded better neuroendocrine modulation of nociception with good cardiorespiratory stability, although it did not offer a more beneficial analgesic effect than systemic medication. In addition, preemptive EPI analgesia using tramadol at 1 and 4 mg/kg of lidocaine was compared with a morphine + lidocaine protocol during orchiectomy and OVH. According to the University of Melbourne’s pain scores, both protocols were similar and had an efficient analgesic effect within 24 h post-surgery. Nevertheless, the synergic effects of the premedication (ketamine + xylazine) might have enhanced the analgesic properties of the drugs [63]. Intraperitoneal (IP) tramadol at 4 mg/kg, combined with lidocaine (8.8 mg/kg IP) following OVH, provided better analgesia, based on scoring methods and lower cortisol and glucose levels at 6 and 3 h post-surgery, respectively; however, the differences were not statistically relevant when compared with animals in other groups [64]. Giorgi et al. [26] investigated rectal tramadol administration and observed that the plasma concentrations were below the effective therapeutic dose, undergoing rapid metabolization to its inactive compounds M2 and M5. Intranasal administration of tramadol (4 mg/kg) after canine OVH presented a similar effect to IV administration of both tramadol and 0.22 mg/kg of methadone, with no considerable alterations in pain scores or cardiorespiratory parameters [49].

Constant rate infusion (CRI) of tramadol has also been used during OVH at a dosage of 22 μg/kg/min (loading dose: 3 mg/kg IV) in combination with dissociative anesthesia. The drug combination decreased anesthetic requirements, shortened recovery times without adverse effects, and provided cardiorespiratory stability [65]. Furthermore, the coadministration of tramadol (4 mg/kg IV) and ketamine and lidocaine by CRI as preemptive analgesia reportedly improved the antinociceptive effect of the opioid following postoperative elective sterilization [66]. In healthy subjects, the combination of a tramadol–lidocaine infusion decreased the minimum alveolar concentration of sevoflurane, in comparison to the use of the opioid alone (2.2% ± 0.3% and 1.7% ± 0.3%, respectively) [67]. Likewise, CRI using tramadol (0.5, 1.0, and 2.0 mg/kg/h) and 5 mg/kg of ketoprofen SC at 50 mg/ml in dogs undergoing laparotomy reduced glucose levels and pain scores during 1 h post-surgery without cardiorespiratory alterations observed on following days [14].

The effect of tramadol on thermal and mechanical acute nociceptive pain has revealed differences in several investigations. In Beagles exposed to thermal stimuli using the tail-flick test, high doses of IV tramadol (6.81 and 10 mg/kg), compared with tapentadol (2.15, 4.64, 6.81 mg/kg IV) and morphine (0.464, 0.681, 1.0 mg/kg), did not induce antinociception and side effects such as ataxia and a short seizure [21]. The dose-dependent antinociception and better analgesic performance of morphine and tapentadol were attributed to their direct action without relying on metabolic activation. Schütter et al. [41] also described the effect of tramadol IV (1 and 4 mg/kg) as questionable to treat acute nociception in Beagles. Although a slight increase in the mechanical threshold was reported, no clinically relevant antinociception was noted at the thermal threshold, and M1 concentrations were below the previously reported concentration [41].

Some authors have suggested the accumulative effect of tramadol after repeated oral dosing as the underlying reason for its effectiveness during chronic or osteoarthritic pain [41]; however, Budsberg et al. [68] reported that dogs orally administered 5 mg/kg/8 h failed to demonstrate a clinical benefit for elbow or stifle joint osteoarticular disease when compared with carprofen (2.2 mg/kg/12 h) and a placebo. Conversely, Benitez et al. [69] achieved similar analgesia to a hydrocodone–acetaminophen protocol following oral administration of tramadol (5–7 mg/kg/8 h) after tibial plateau leveling osteotomy. Piras et al. [70] determined that cimicoxib and tramadol have similar analgesic potency for long-term analgesia for the same procedure. However, treatment with a selective COX-2 inhibitor conferred superior functional improvements. Therefore, tramadol may be unsuitable as monotherapy for osteoarthritic pain. Accordingly, it has been established that tramadol (at 3–5 mg/kg every 8 h for 4 weeks), in addition to NSAIDs, increases the peak vertical force in 50% of the subjects, a similar percentage to dogs treated with gabapentin (61%); hence, both drugs can be recommended in this clinical condition and as a part of multimodal analgesia [71]. In a controlled trial conducted by Malek et al. [72], animals with naturally occurring hip osteoarthritis received tramadol at 4 mg/kg/8 h orally for 2 weeks. The animals showed improved physical parameters, pain, and mobility in all treatment groups. The authors revealed two significant findings regarding tramadol: i) the improved pain interference was superior to the improvement in pain severity; and ii) decreasing plasma tramadol concentrations following 2 weeks due to the impaired biotransformation in domestic canines. Therefore, tramadol treatment for chronic pain management is still questionable [72]. The prevalence of low M1 and high M2 concentrations was reported by Giudice et al. [19] in dogs with neuropathic pain due to degenerative lumbosacral stenosis, where 50% of patients did not show a reduction in pain scores after 1 week of oral dosage (3 mg/kg/8 h) together with prednisolone; accordingly, the treatment protocol was replaced with oral gabapentin. In a recent work by the same author [73], buprenorphine (0.02 mg/kg IM), a partial mu-opioid receptor agonist, offered better postoperative analgesia in dogs subjected to hemilaminectomy when compared with 3 mg/kg IM tramadol; the study also revealed that tramadol might be a better option for follow-up treatment. Based on these findings, tramadol seems appropriate for reducing lumbosacral pain to some degree, but some dogs might fail to respond despite high doses and a shortened dosing time. Ripplinger et al. [74] supported this statement after evaluating 180 dogs undergoing vertebral surgery, where 15% of 46 dogs receiving tramadol (3 to 8 mg/kg SC) revealed a higher percentage of persistent pain than other opioids (morphine and methadone) (15%, 4.8%, and 3.3%, respectively). The authors attributed this finding to pharmacokinetics and suggested that tramadol should not be employed as monotherapy or first-line therapy for long-term orthopedic pain management.

In other procedures such as surgical resection of cutaneous tumors, the effects of preemptive tramadol administration (3 mg/kg every 8 h), orally for 48 h before surgery, were compared with carprofen (2.2 mg/kg/12 h) and no preemptive analgesia, in combination with preoperative hydromorphone [75]. According to pain scores, there was no difference between animals that received and did not receive preemptive analgesia, and this could be due to the premedication time, surgical procedure, and the surgeon itself. In mastectomies and OVH, tramadol (5 mg/kg IM) did not offer superior analgesia when compared with methadone (0.5 mg/kg IM), another opioid. The group medicated with methadone required less complementary analgesia and showed lower drug consumption with better postoperative pain scores [76]. Using the same surgical procedure, Reis et al. [77] determined that the addition of tramadol (2 and 4 mg/kg EPI) to an anesthetic protocol of tramadol + propofol + levobupivacaine potentiated and reduced the propofol requirement while providing adequate analgesia, with vascular stability but without differences when compared with propofol + levobupivacaine alone. Furthermore, the analgesia provided by oral tramadol (12 mg/kg/24 h) (combined with an anesthetic protocol of methadone, bupivacaine, and carprofen) for lateral thoracotomy exhibited higher pedometric activity than patients receiving fentanyl transdermal patches [78].

Accordingly, available scientific evidence indicates that tramadol may be an acceptable alternative for dogs only when administered with other analgesic drugs [48]. In canines, its efficacy depends on hepatic activation and is influenced by genetic or pharmacological factors. Moreover, the administration of CYP substrates or inhibitors can increase opioid bioavailability, thereby prolonging and intensifying analgesic and adverse effects [38]. For example, ketoconazole and cimetidine are inhibitors of CYP3A, an enzyme that produces M2. Their use with tramadol can reduce first-pass metabolism and the formation of M2 and M5 from M1 metabolite [79]. On the contrary, phenobarbital has the opposite effect and reduces the analgesic efficacy of this opioid by altering drug metabolism and M1 concentrations [38].

## Therapeutic Efficacy of Tramadol in Cats

In cats, opioids are not always considered first-choice drugs owing to the reduced glucuronidation activity that can increase the risk of toxicity when doses and intervals are not administered appropriately [80]. The main side effects following high doses of tramadol are sympathetic responses such as mydriasis, hypertension, tachycardia, and an increase in adrenal hormone concentration (e.g., NE, epinephrine, dopamine, met-enkephalin). Dysphoria, excitement, anxiety, and vocalizations have also been reported [81]. Nevertheless, tramadol is a potential alternative to NSAIDs, drugs not well-tolerated or contraindicated in some cases, and for treating certain pre-existing diseases [7]. The bitter taste of tramadol is also considered a limiting factor in cats [51].

The effect of tramadol in surgical settings as perioperative analgesia has been examined in various studies in recent years. Teixeira et al. [22] evaluated 2 mg/kg/8 h SC tramadol alone and in combination with IV dipyrone at different doses (25 mg/kg every 8, 12, or 24 h) in patients undergoing OVH. Biochemical, hematological, and behavioral parameters were assessed. The authors revealed that cats receiving dipyrone + tramadol required less complementary analgesic; however, it did not offer a superior analgesic combination than the use of the opioid alone [22]. Likewise, IV administration of 3 mg/kg tramadol during OVH and orchiectomy, along with meloxicam (0.05 mg/kg SC), revealed that 89.4% of the animals presented only mild pain [82]. In a recent work from Bovo et al. [83], the IM administration of tramadol (2 mg/kg) produced a similar analgesic efficacy to morphine (0.5 mg/kg), with 40% of patients requiring rescue analgesia in both groups; however, tramadol induced a lower degree of sedation than the pure agonist.

A comparative study between IM tramadol at 2 and 4 mg/kg, and IM pethidine (6 mg/kg) determined the dose-dependent analgesic effect of tramadol [53]. At the highest dose, serum cortisol values and pain levels were lower, and the animal did not require additional analgesia. Associations of tramadol (2 mg/ kg IM) and midazolam (0.2 mg/kg IM) as pre-anesthetic protocol for OVH were compared with dexmedetomidine. The tramadol group had fewer secondary effects (nausea and vomits), a faster recovery time, and better cardiovascular stability than the alpha-2 adrenergic agonist [84]. Furthermore, given the absence of cardiorespiratory changes and physiological parameters within normal ranges, Evangelista et al. [53] suggested that tramadol is a reliable perioperative drug for cats undergoing OVH at the recommended doses. In addition, Li et al. [85] investigated a mixture of tiletamine–zolazepam–xylazine–tramadol during sterilization. This protocol enhanced the induction of the anesthesia and the antinociceptive effect of the drugs without cardiopulmonary side effects. A study by Nascimento et al. [86] mentioned the addition of tramadol at 2 mg/kg IM as rescue analgesia in patients subjected to laser acupuncture and electroacupuncture therapies before OVH. Despite its analgesic effect, 3 out of 30 cats required additional meloxicam when the opioid failed to improve postoperative pain scores. On the contrary, Martins et al. [87] determined that perioperative tramadol (2 mg/kg IM) along with acepromazine and tiletamine/zolazepam conferred a higher analgesic effect, with lower heart rate and a stable respiratory rate in prepubertal cats undergoing OVH.

Experimentally, tramadol (1 mg/kg) and acepromazine (0.1 mg/kg), both SC, were evaluated on pressure and thermal thresholds. Tramadol monotherapy offered limited effects on nociceptive thermal and pressure stimuli, but improvements were observed when administered with acepromazine [80]. In many cases, the neuroleptoanalgesia afforded by acepromazine increases the weak analgesic effect of the opioid. In particular, this pre-anesthetic association has also been considered a suitable option for ophthalmic surgery in cats [88], with benefits such as unaltered intraocular pressure and sustained mydriasis when co-administered with tramadol (3 mg/kg IM), distinct from acepromazine alone (0.05 mg/kg IM) [89]. The analgesic potency of 2 mg/kg IM tramadol or EPI tramadol co-administered with lidocaine (3 mg/kg EPI) was also evaluated in terms of painful mechanical stimuli following pressure application from hemostatic forceps in the skin of several body regions. Both administration routes demonstrated a short anesthetic onset and similar motor blockade duration without cardiorespiratory changes. Regarding analgesia, EPI tramadol with lidocaine produced more prolonged analgesia than IM administration (120 ± 31 min *vs.* 71 ± 17 min, respectively) [37].

In osteoarticular diseases associated with chronic pain, nociception, inflammation, central and peripheral sensitization, hyperalgesia, allodynia, and a reduction in mobility, tramadol is recommended despite necessitating prolonged treatment periods [20]. Clinical trials in geriatric cats with osteoarthritis medicated with different dosages (1–4 mg/kg) of oral tramadol indicate that 2 mg/kg/12 h for 5 days increased the level of activity and the global quality of life; however, adverse effects such as euphoria, dysphoria, sedation, hyporexia, and diarrhea were present at high doses (4 mg/kg) [43]. Monteiro et al. [20] observed a favorable response in mobility, biomechanical aspects, and hypersensitivity in patients medicated with oral tramadol at 3 mg/kg/12 h for 19 days and three-non-treatment months during the therapy. Conversely, another study evaluated the analgesic efficacy of oral tramadol (3 mg/kg/12 h) with oral transmucosal meloxicam (approximately 0.05 mg/kg/24 h) for 25 days. Animals administered tramadol did not show a clinical benefit over meloxicam alone, except in the case of central hypersensitivity [34]. In addition, some cats showed mydriasis, depression, hyporexia, hypersalivation, and vomiting, although these reactions could not be attributed to a specific treatment.

Tramadol has also been used for treating polytraumatized cats [90], and it is known to significantly diminish central sensitization in neuropathic pain. Additionally, transdermal patches of tramadol exist, but there is no literature on its use in domestic species [91].

## Clinical Pharmacology of Tapentadol

**Figure 2.**
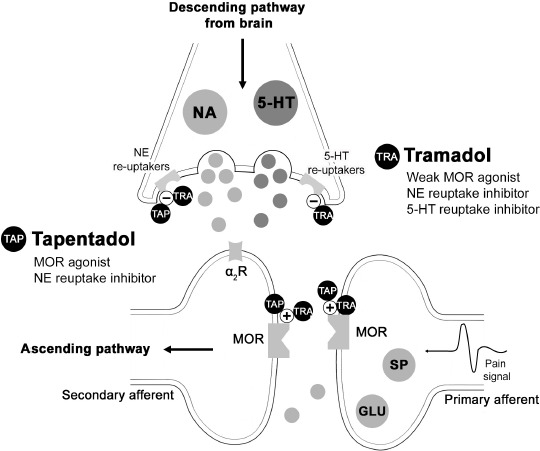
Mechanism and sites of action of the opiods tramadol and tapentadol. α_2_R = alpha-2 receptors, GLU = glutamate, SP = substance P.

Tapentadol was formulated based on morphine, tramadol, and its active M1 metabolite building, but exists as a single enantiomer commercially available for oral administration [92,93]. It is a central acting atypical opioid with agonism toward MOR while simultaneously inhibiting neuronal reuptake of NE without any clinical evidence of serotonergic activity, unlike tramadol [21,94] (Fig. 2). It blocks modulation and perception of the nociceptive pathway, acting on the ascending tracks via MOR agonism [93]. The affinity of tapentadol for MOR, compared to morphine, is approximately 50-fold below but 50-fold to 120-fold greater than tramadol. Thus, it has an analgesic potency almost identical to morphine, with adequate oral absorption, minimal physiological effects, and a similar plasma concentration in animals and humans [94,95].

The pharmacokinetic profile of tapentadol makes it an attractive analgesic option when compared with tramadol [96], especially in those species where active M1 concentrations are negligible, such as dogs. Following IV (20 mg) and oral (200 mg single dose) administration in dogs, measurable levels of plasma concentration were detectable for up to 6 h and 15–240 min, respectively [97], with a terminal half-life of 0.5–1 h [98]. In cats, the parenteral route achieved a bioavailability above 90% with a short elimination half-life (2–3 h) [91]. On the contrary, oral administration revealed a decreased bioavailability due to reduced glucuronidation (Table 3) [95,97,99].

The advantage of tapentadol administration as an active compound is the safety and efficacy that does not warrant CYP450 liver enzyme-dependent metabolic activation; this reduces the inter-individual analgesic variabilities due to genetic polymorphisms and decreases the possibility of adverse interactions when administered with other drugs [18,97]. Moreover, it is considered a good option for patients with mild or moderate hepatic or renal impairment without affecting its analgesic effect [92].

**Table 3.** Comparison of mean ± SD values for some tapentadol pharmacokinetic variables in dogs and cats.

Species	Route and dose	Parameters (units)	Mean ± SD values	References
Dogs	IV (50 mg/dog)	*F*% Vd (ml/kg) Cl (ml/min/kg) *T*_max_ (min) *C*_max_ (ng/ml)	ND 5,917–7,236 59.8–116.6 ND ND	[97]
	IV (30 mg/kg)	*F*% Vd (ml/kg) Cl (ml/min/kg) *T*_max_ (h) *C*_max_ (ng/ml)	ND ND ND 3.5 ± 1.2 31.0 ± 7.5	[95]
	IV (20 mg/kg)	*F*% Vd (ml/kg) Cl (ml/min/kg) *T*_max_ (h) *C*_max_ (ng/ml)	ND ND ND 2.4 ± 1.2 19.7 ± 5.5	[95]
	IV (10 mg/kg)	*F*% Vd (ml/kg) Cl (ml/min/kg) *T*_max_ (h) *C*_max_ (ng/ml)	ND ND ND 2.7 ± 0.9 10.2 ± 1.8	[95]
	Oral (200 mg/dog)	*F*% Vd (ml/kg) Cl (ml/min/kg) *T*_max_ (min) *C*_max_ (ng/ml)	4.4 ± 2.3 7,024–55,358 1,138–2,384 47.5 ± 6.1 240–3,640	[97]
Cats	IV (5 mg/kg)	*F*% Vd (ml/kg) Cl (ml/min/kg) *T*_max_ (h) *C*_max_ (ng/ml)	ND 8.79 ± 1.97 35.60 ± 7.05 ND ND	[99]
	IM (5 mg/kg)	*F*% Vd (ml/kg) Cl (ml/min/kg) *T*_max_ (h) *C*_max_ (ng/ml)	93.93 ± 9.91 7.53 ± 2.95 37.85 ± 5.68 0.25 ± 0.26 1,406 ± 779	[99]
	SC (5 mg/kg)	*F*% Vd (ml/kg) Cl (ml/min/kg) *T*_max_ (h) *C*_max_ (ng/ml)	90.01 ± 6.52 7.06 ± 2.10 40.13 ± 9.97 0.63 ± 0.31 906 ± 356	[99]

*F*% = bioavailability, ND = not determined, Vd = apparent volumen of distribution at steady-state, Cl = clearance, *T*_max_ = time of maximum plasma concentration, *C*_max_ = maximum plasma concentration.

The metabolism is mainly through phase II glucuronidation, with sulfation playing a minor role and minimal contribution from CYP enzymes [18,95]. Apparently, the metabolites produced do not provide analgesia, resulting in fewer side effects (e.g., sialorrhea or sedation) [97]; however, some studies have reported an accumulative effect after repeated dosing [95].

The antinociceptive effect of the marked NE reuptake inhibition on terminal endings and the descending pain pathway suppresses the noxious signaling by stimulating alpha-2 adrenergic receptors in the nociceptive fibers. The inhibitory mechanism of NE is reportedly predominant during the modulation of chronic and neuropathic pain [17,93]. Likewise, because tapentadol does not use serotonergic pathways, there are no reports of serotonin syndrome in animals, and the possibility of developing this toxicity is reduced [18]. However, a study has documented serotonin-related toxicity when administered in association with tramadol, duloxetine, venlafaxine, amitriptyline, sertraline, desvenlafaxine, and escitalopram, but a clear correlation cannot be established in dogs and cats [100]. Conversely, unlike tramadol, opioid antagonists like naloxone cannot suppress the effects of tapentadol, corroborating the dual mechanism of this drug [17].

It is generally well tolerated, but it may cause excitability in cats and depression, panting, and salivation in dogs. Disadvantages include antimuscarinic activity and low oral bioavailability, although in animals with deficient glucuronidation, such as cats, tapentadol bioavailability may be enhanced [97]. Similar to tramadol, tapentadol is bitter and can be limiting in this species [91].

It can treat mild to severe pain when animals need prolonged analgesia [99]. In chronic pain, tapentadol is administered as part of multimodal protocols combined with NSAIDs. To date, there are no recommended dosages in companion animals, but clinical doses mentioned by Gaynor and Muir [98] range between 5 and 10 mg/kg, every 12 or 8 h, with a half-life of 0.5–1 h in dogs.

Studies evaluating the analgesic effect of tapentadol in dogs and cats are limited [101]. Moreover, recent experimental animal models have shown good antinociception associated with mechanical stimulation and acute orofacial pain in adult rats medicated with tapentadol at 2 and 5 mg/kg [102]. In another study, tapentadol demonstrated a dose-dependent effect and inhibited the electrophysiological neuronal activity in the *locus coeruleus* in a rat model of diabetic polyneuropathy [103]. In addition, tapentadol enhances the inhibitory activity of NE descending pathways and prevents nociceptive signaling at the spinal cord of rats with osteoarthritis [104]. These authors also mentioned that alpha-adrenergic drugs such as yohimbine and atipamezole could block tapentadol action. Tapentadol has also exhibited a dose-dependent antinociceptive effect (approximately 99%) in rodents without causing gastric alterations [105], whereas a synergistic effect was observed when co-administered with NSAIDs (e.g., diclofenac and ketorolac) in rats assessed using the writhing test [106].

## Experimental Research and Future Expectations for the Use of Tapentadol in Dogs and Cats

As stated earlier, investigations assessing tapentadol and its analgesic effects in small animals are scarce, but some studies have elucidated its potential in veterinary medicine. In dogs, Howard et al. [95] reported the pharmacokinetics and pharmacodynamics of oral tapentadol (at 10, 20, and 30 mg/kg). The highest plasma concentration was detected approximately 2.7–3.5 h after administration, with rapid absorption and no side effects [95]. On the contrary, high doses (6.8 mg/kg IV), produced sedation, ataxia, sialorrhea, and diarrhea in Beagle dogs [21]. In a similar work, Kögel et al. [21] used the tail-flick model test to assess acute nociceptive pain, comparing the analgesic potential of IV tramadol, morphine, and tapentadol (2.15, 4.64, and 6.8 mg/kg, respectively). Unlike tramadol, morphine and tapentadol induced antinociception through direct action on MOR without the need for metabolic activation, indicating their potential for reliable analgesia in clinical settings.

In dogs with orthopedic pain due to naturally occurring cranial cruciate ligament rupture, tapentadol (30 mg/kg orally and single dose) lowered subjective lameness scores 4 h after administration [94]; however, no significant improvement in the gait analysis was observed, which was evaluated by a pressure-sensitive gait system. As the plasma level required to achieve adequate analgesia in dogs remains unknown, Kieves et al. [94] concluded that the ranges obtained in this study (9–49 ng/ml) might need to be higher, along with repeated doses, to achieve the therapeutic efficacy with long-term treatment; however, further studies are needed.

Concerning tapentadol in cats, Doodnaught et al. [92] evaluated the thermal antinociception of oral dosages of 5.7 and 11.4 mg/kg in healthy cats. The thermal threshold and skin temperature rise for up to 2 h after administering the highest dose. The duration of thermal antinociception was similar between tapentadol at 11.4 and 0.02 mg/kg of buprenorphine IM, with salivation as the only documented side effect [92]. This observation is consistent with the results by Lee et al. [99], where 5 mg/kg IV caused salivation, agitation, and panting. In this same study, the authors determined the bioavailability of parenteral IM and SC tapentadol (94% and 90%, respectively).

Some authors have carried out pharmacokinetic and toxicological investigations in other species, including recent studies in Amazon parrots [107] and laying hens [108]. In parrots, the short half-life (24.8 min) following one dose of 30 mg/kg per oral (PO) indicated its limited use in this species; in hens, only IV administration (1 mg/kg) exhibited minimum therapeutic efficacy when compared with PO administration (5 mg/kg), showing a half-life of 0.9 h and a low bioavailability. Additional studies have been carried out in red-eared slider turtles [109], yellow-bellied slider turtles [110], Wistar rats [111], and goats [112]. Antinociceptive effects have been evaluated in rabbits [113], rats, and mice [105], all of which demonstrated adequate analgesia at 5 mg/kg IV dosages.

As previously reported, analgesic assessments of tapentadol in companion animals are insufficient to reach a definitive conclusion before recommending its use in veterinary practice. Additionally, some limitations in this study and the selected papers should be noted. Given the inclusion criteria and previously mentioned publications, all works that employed tramadol and tapentadol as an analgesic or antinociceptive therapy in different pain-related settings in dogs and cats were included. The sample size of animals used in selected articles was not deemed an exclusion/inclusion criterion in the present review; however, some reported data included clinical trials with minimal subjects, leading to a false absence of statistical differences between groups or subjects. Nonetheless, these findings contribute to critical and scientific knowledge that needs to consider these factors. Regarding tapentadol, the limited available data for an analgesic protocol in veterinary medicine is, *per se*, a limitation to objectively propose or reject the application of this drug; hence, animal models and nociception tests can help veterinarians to broaden their view on this analgesic alternative.

To summarize the characteristics of tramadol and tapentadol, the doses and mechanisms of action are presented in Table 4 [81,91,114]. Table 5 [16–18,20,25,26,31,35,93,95,99] presents a brief comparison of relevant pharmacological aspects and differences between tramadol and tapentadol in dogs and cats.

## Serotonin Syndrome Induced by Tramadol and Serotonin Reuptake Inhibitors

It has been established that tramadol possesses a non-opioid mechanism of action that promotes the inhibition of 5-HT reuptake, increasing its extracellular levels in the brain [115]. 5-HT is stored primarily in the presynaptic nerve terminals, in enterochromaffin cells, and within platelets [116]. It is an inhibitory neurotransmitter used in animals to treat separation anxiety, canine cognitive dysfunction, and compulsive disorders, as well as an adjuvant for pain control [116].

**Table 4.** Dosage and pharmacological characteristics of tramadol and tapentadol [81,91,114].

Drug	Species	Doses	Route	Mechanism of action	Adverse effects
Tramadol	Dogs	4–10 mg/kg/6–8 h	IV, IM, PO	Agonism to μ-receptors Inhibits monoamine reuptake (NE and 5-HT)	Tremors, myoclonus, tachycardia, agitation, excitement, hypertension, and seizures (signs of serotonin toxicity)
	Cats	1–4 mg/kg/12–24 h	IM, IV, PO		
Tapentadol*	Dogs	2–30 mg/kg	IV, PO	μ-Opioid receptor agonist NRI	Salivation, agitation, panting, mydriasis, euphoria, and sedation
	Cats	5–10 mg/kg q 12 h	IV, IM, PO, SC		

PO = oral.

* doses taken from the available literature.

**Table 5.** Summary of some pharmacological differences between tramadol and tapentadol in dogs and cats [16–18,20,25,26,31,35,93,95,99].

	Tramadol		Tapentadol	
	Dogs	Cats	Dogs	Cats
Administration routes	IV, IM, PO, SC		IV, PO	
Absorption	Intestinal		Intestinal	
Oral bioavailability	60%–83%	60%–90%	4.4%	Unknown
Plasma concentration (ng/ml) (M1)	146–449	366–850	240–3640	906–1,406
Metabolism	Hepatic		Hepatic	
Main metabolites	M2	M1 and M5	Tapentadol-O-glucoronide	
Active metabolite	M1		Active parent compound	
Terminal half-life (M1)	1–2 h	4–6 h	2–5 h	1–3 h
Excretion	Urine and feces		Urine and feces	
Interactions	Combination with a SSRI can cause serotonin syndrome Naloxone, alpha-2 antagonist, and 5-HT partially blocks		The antagonist atipamezole, and yohimbine could potentially block the effect of tapentadol	
Differences between species	CYP polymorphism affects its analgesic efficacy in dogs		It does not rely on metabolism to produce its therapeutic effects, reducing the inter-species differences.	

SSRI = Selective serotonin reuptake inhibitor.

**Table 6.** Drugs that can contribute to serotonin syndrome, in addition to another serotoninergic *drug,* or *overdosing* [51,117].

Type or drug	Examples
Analgesic	Tramadol
Selective 5-HT reuptake inhibitors	Citalopram, escitalopram, fluoxetine, fluvoxamine, nefazodone, paroxetine, sertraline, trazodone.
Tricyclic antidepressant	Amitriptyline, clomipramine, desipramine, dosulepin, doxepin, imipramine, nortriptyline, opipamol, trimipramine.
NRI and 5-HT reuptake inhibitors	Desvenlafaxine, duloxetine, milnacipran, sibutramine, venlafaxine.
Atypical antipsychotics	Aripiprazole, asenapine, bupropion, clozapine, lurasidone, olanzapine, quetiapine, risperidone, ziprasidone.
Selective MAO inhibitors	Brofaromine, metralindole, minaprine, moclonemide, pirlindol, toloxatone, rasagiline, selegiline, amitraz.
Non-selective MAO inhibitors	Hydralazine, isocarboxazid, isoniazid, phenelzine, phenoxypropazine, safrazine, caraxazona, furazolidone, linezolid.
Other	5-HT, chlorpheniramine, dextrometophan, lithium, metoclopramide, ondansetron, tryptophan.

MAO = monoamine oxidase.

**Table 7.** Clinical signs of serotonin syndrome in companion animals [115,117].

Body system	Clinical signs
Cardiovascular	Tachycardia, bradycardia, arrhythmia, hypertension
Respiratory	Tachypnea
Nervous system	Sedation, lethargy, hyperexcitability, temporary blindness, nystagmus
Mental status	Confusion, agitation, excitement, vocalizations, aggression, coma
Neuromuscular	Hyperreflexia, muscle spasms and hyperthermia, trembling, rigidity, convulsions, recumbency, weakness, hyperesthesia
Gastrointestinal	Hypersalivation, nausea, vomiting, diarrhea, abdominal pain
Metabolic	Fever or hyperthermia with diaphoresis

The concomitant administration of drugs that can increase the concentration of 5-HT, such as tramadol and other medication listed in Table 6 [51,117], potentiates the risk of developing serotonin syndrome [115].

This syndrome affects dogs and cats, typically occurs within the first 30 min to 2 h post-ingestion and demonstrates multiple clinical signs in patients (Table 7) [115,117].

Indrawirawan and McAlees [118] published a case of accidental tramadol overdose (80 mg/kg orally) in a cat. The animal exhibited agitation, hypersalivation, hypertension, tachycardia, abdominal pain, head tremors, myoclonus, hyperreflexia, paresis, disorientation, and altered mental status. In cats, deficient hepatic methylation and glucuronidation enhance the predisposition to severe adverse effects [50]. Toxicity has also been recorded in dogs, with similar signs mentioned in the case report [50].

The initial treatment requires the administration of serotonergic antagonists such as cyproheptadine (1.1 mg/kg q 1–2 h PO) or chlorpromazine (3 mg/kg) [119]. Supportive therapy includes phenobarbital or propofol in case of seizures (benzodiazepines can increase arousal), beta-blockers (propranolol), muscle relaxants (methocarbamol), antihypertensives and fluid therapy (crystalloids), physical methods to reduce hyperthermia, tracheal intubation if neuromuscular paralysis occurs, and cardiorenal, electrolyte and glucose monitorization [117,119].

The main complications include rhabdomyolysis, myoglobinuria, intravascular coagulation, renal insufficiency, respiratory and CNS depression, and even death [115]. For preventing serotonin syndrome, the combination of serotoninergic drugs should be avoided and administered doses need to be regulated, especially in individuals with reduced liver metabolism [120].

## Conclusion

Tramadol and tapentadol are analgesics administered to manage pain in dogs and cats. These drugs have opioid and non-opioid mechanisms that influence their analgesic effect and efficacy in companion animals. Tramadol, a pain reliever widely employed in animals, is dependent on liver metabolism to produce the principal active M1 metabolite. Compared with cats, dogs differ in their capacity to metabolize M1 due to polymorphisms in hepatic enzymes, which affect the analgesia induced by tramadol. On the contrary, the novel opioid tapentadol has potential advantages over tramadol as it does not depend on hepatic biotransformation, acts through NE reuptake inhibition, and demonstrates stronger MOR agonism; however, to date, the investigations assessing the analgesic efficacy of tapentadol remain limited in veterinary medicine. Given these points and available evidence on both drugs, tramadol appears to be a more suitable therapeutic option for cats and, preferably, should be administered as a multimodal analgesia protocol in both species, particularly dogs. Tapentadol might have a superior analgesic profile in animals, but the effectiveness of this opioid needs to be further clarified before recommending its use for managing acute and chronic pain in dogs and cats.

## List of Abbreviations

CNS: central nervous system; COX-2: cyclooxygenase 2; CRI: constant rate infusion; CYP: cytochrome P450; EPI: epidural; h: hours; 5-HT: serotonin; IM: intramuscular; IV: intravenous; M1: O-desmethyltramadol; M2: N-desmethyltramadol; M5: N,O-didesmethyltramadol; MOR: μ-opioid receptor; NE: norepinephrine; NMDA: N-methyl-D-aspartate; NRI: norepinephrine reuptake inhibitor; NSAIDs: non-steroidal anti-inflammatory drugs; POR: CYP oxidoreductase; SC: subcutaneous; TRPV1: transient receptor potential vanilloid 1; UDP: uridine diphosphate; UGT: UDP-glucuronyltrasferase; OVH: ovariohysterectomy.
